# Comparative Reconstruction of Intrinsic Apoptosis in Clade V Nematodes Reveals a Conserved Execution Module and Heterogeneous Regulatory Architecture

**DOI:** 10.34133/csbj.0239

**Published:** 2026-11-13

**Authors:** Marc Kaethner, Neil D. Young, Brad E. Sleebs, Erinna F. Lee, Walter D. Fairlie, Robin B. Gasser

**Affiliations:** ^1^ Department of Veterinary Biosciences, Melbourne Veterinary School, The University of Melbourne, Parkville, VIC, Australia.; ^2^ Walter and Eliza Hall Institute of Medical Research, Parkville, VIC 3052, Australia.; ^3^ Department of Biochemistry and Chemistry, School of Agriculture, Biomedicine and Environment, La Trobe University, Bundoora, VIC, Australia.; ^4^ La Trobe Institute for Molecular Science, La Trobe University, Bundoora, VIC, Australia.

## Abstract

Intrinsic apoptosis is a form of programmed cell death, yet its regulatory architecture varies across Metazoa. In parasitic nematodes, this pathway is of particular interest because conserved components could represent candidates for anthelmintic intervention. However, pathway-level resolution across nematodes remains limited, and computational homology alone does not establish conserved biological function. Here, we assessed the evolutionary conservation of candidate intrinsic-apoptosis-associated proteins across a resource-defined set of clade V nematodes. Building on recent pathway reconstruction in the parasitic nematode *Haemonchus contortus*, we combined orthology inference, genome-guided homology searches, and structure-informed approaches. Homologs corresponding to the BCL-2-like, APAF-1-like, and CASP-3-like execution module were recovered from almost all species and showed high structural similarity among parasitic clade V nematodes. For BCL-2-like and APAF-1-like proteins in particular, structural similarity was greater among parasitic nematodes than between parasite proteins and their corresponding host homologs; these global comparisons inform target prioritization but do not establish drug selectivity. Regulatory components showed heterogeneous evolutionary patterns: FBXO-11-like homologs were comparatively well conserved, and PUM-1/PUM-2-like homologs showed intermediate conservation, whereas p53-like and especially EGL-1-like homologs were more divergent and variably detectable. No additional CED-13-like homolog was detected using sequence- and genome-based searches or, where structural coverage was available, structure-based searches. These findings support a comparatively conserved execution module embedded within a heterogeneous regulatory architecture and provide a framework for prioritizing apoptosis-associated proteins for experimental investigation in anthelmintic discovery.

## Introduction

Programmed cell death is a defining feature of multicellular life, underpinning development, tissue homeostasis, and cellular responses to stress [1]. Intrinsic apoptosis, in particular, is a relatively conserved regulatory system across Metazoa, classically defined in vertebrates by mitochondrial outer membrane permeabilization (MOMP), cytochrome *c* release, and assembly of the apoptosome [1]. This pathway is controlled by interactions among BCL-2 family proteins, adaptor proteins, and caspases and has been characterized at the molecular level in some vertebrates (e.g., humans) and invertebrates (e.g., flatworms, roundworms, and insects) [1]. However, comparative studies have revealed that the architecture and regulation of intrinsic apoptosis can vary substantially across phyla, indicating that conserved execution mechanisms may be embedded within divergent lineage-specific regulatory frameworks (cf. [2]).

In parasitic helminths, understanding apoptosis is of particular interest because conserved components of this pathway could represent targets for therapeutic intervention [3]. In platyhelminths, such as schistosomes, a BCL-2-regulated apoptosis pathway has been described that closely resembles the vertebrate system. Here, apoptosis is initiated by multiple BH3-only proteins and is restrained by prosurvival BCL-2-related proteins, and BAX/BAK proteins execute MOMP, triggering cytochrome *c*-dependent APAF-1 apoptosome assembly and caspase activation [3,4]. By contrast, early genetic studies in the free-living nematode *Caenorhabditis elegans* defined a simplified pathway in which the BCL-2-like protein CED-9 directly inhibits the adaptor APAF-1-like protein CED-4 [5]. When CED-9 engages the BH3-only protein EGL-1 or CED-13, CED-4 is released, enabling the apoptosome to assemble, leading to the activation of the CASP-3-like protein, CED-3, without a requirement for a BAX/BAK-like protein and cytochrome *c*-dependent apoptosome assembly [6,7]. These observations suggest that intrinsic apoptosis in nematodes operates through a mechanistically distinct, MOMP-independent mode of regulation. In *Caenorhabditis elegans*, DNA-damage-induced germline apoptosis is further regulated upstream by the p53-like transcription factor CEP-1 [8], which induces the expression of *egl-1* and *ced-13* [6,9]. In addition, the FBXO-11-like protein DRE-1 inhibits CED-9 [10] and the PUM-1/PUM-2-like protein PUF-8 activates or inhibits apoptosis by binding to *ced-3* and *ced-9* messenger RNAs, thereby repressing their expression [11,12].

Recent comparative genomic analyses have demonstrated that the components and regulation of apoptosis pathways in nematodes are diverse [2]. A phylum-wide survey of BCL-2 family and APAF-1 homologs revealed extensive variation in gene complement, domain architecture, and structural features across clades, including the expansion of BCL-2 family proteins in some taxa and the presence of WD40-repeat motifs, critical for engaging MOMP products such as cytochrome *c*, in APAF-1 homologs of basal lineages [2]. These findings established that the *Caenorhabditis elegans* pathway does not generalize to all nematodes and highlighted the need for pathway-level reconstruction in parasitic species. Building on this framework, we recently reconstructed candidate components of the intrinsic apoptosis pathway in the clade V parasitic nematode *Haemonchus contortus* by integrating genome-wide homology searches, structural modeling, and developmental transcriptomic and proteomic analyses [13]. That study identified homologs corresponding to the BCL-2:APAF-1:CASP-3 execution module and evidence consistent with regulatory divergence, including stage-specific transcription and posttranscriptional control [13]. These computational and molecular associations provide a pathway model for comparative testing but do not by themselves establish equivalent protein functions across species.

Despite these advances, it remains unclear which features of the intrinsic apoptosis pathway reconstructed in *Haemonchus contortus* extend across clade V nematodes, particularly parasitic taxa of major medical and agricultural importance (cf. [14]). A key unresolved question is whether candidate pathway components show patterns of evolutionary and structural conservation that justify their prioritization for experimental study while recognizing that homology and structural similarity do not establish conserved function. Addressing this question requires comparative genomic analysis across multiple taxa, combined with approaches capable of detecting highly divergent regulators that are not readily identifiable using standard sequence-based homology methods [2]. Here, we extend comparative pathway reconstruction across a resource-defined set of clade V nematodes using orthology inference, genome-guided homology searches, and structure-informed analyses. By integrating sequence-, genome-, and structure-based evidence within a phylogenetic framework, we aim to define patterns of conservation and divergence among candidate intrinsic-apoptosis-associated proteins and assess their value for prioritizing experimental investigation in anthelmintic discovery.

## Materials and Methods

### Taxon selection and inference of apoptosis pathway homologs

To reconstruct candidate intrinsic apoptosis pathway components across clade V nematodes, species were selected for which annotated genome assemblies with a scaffold N50 >1 Mb were available as of 2026 February 2. This resource-driven criterion was not intended to provide phylogenetically balanced sampling across clade V; *Caenorhabditis* is overrepresented because substantially more high-quality genomic resources are available for this genus than for many other lineages. The dataset comprised 13 free-living species (*Allodiplogaster sudhausi*, *Caenorhabditis bovis*, *Caenorhabditis briggsae*, *Caenorhabditis elegans*, *Caenorhabditis inopinata*, *Caenorhabditis nigoni*, *Caenorhabditis remanei*, *Caenorhabditis tropicalis*, *Diploscapter coronatus*, *Mesorhabditis belari*, *Oscheius tipulae*, *Pristionchus exspectatus*, and *Pristionchus pacificus*) and 8 parasitic species (*Ancylostoma duodenale*, *Angiostrongylus vasorum*, *Cylicocyclus nassatus*, *Haemonchus contortus*, *Necator americanus*, *Nippostrongylus brasiliensis*, *Ostertagia ostertagi*, and *Teladorsagia circumcincta*). The accession codes of all genomes and proteomes used in this study are listed in Table 1 and Table S1. Proteome datasets were obtained from WormBase ParaSite [15,16] or the National Center for Biotechnology (NCBI), except for *Teladorsagia circumcincta*, for which data were published but not publicly accessible at the time and were provided by Dr S. R. Doyle (Wellcome Sanger Institute, Hinxton, UK). Genome and proteome completeness was assessed using BUSCO and is reported in Table S1. To standardize datasets, only the longest transcript per gene was retained using utilities in OrthoFinder v3.1.2 [17,18].

**Table 1.** Clade V nematodes included in this study. Listed are species, lifestyle, genome assembly accession, and reference. A more detailed table including scaffold N50, genome and proteome BUSCO completeness, download source, and number of modeled protein structures is given in Table S1.

Species	Lifestyle	Genome assembly	Reference
*Ancylostoma duodenale*	Parasitic	GCA_050947105.1	[47]
*Angiostrongylus vasorum*	Parasitic	GCA_018806985.1	[48]
*Cylicocyclus nassatus*	Parasitic	GCA_958299035.1	[49]
*Haemonchus contortus*	Parasitic	GCA_041937105.1	[50]
*Necator americanus*	Parasitic	GCF_031761385.1	[16,51,52]
*Nippostrongylus brasiliensis*	Parasitic	GCA_030553155.1	[53]
*Ostertagia ostertagi*	Parasitic	GCA_964213955.1	[54]
*Teladorsagia circumcincta*	Parasitic	GCA_964273815	[55]
*Allodiplogaster sudhausi*	Free-living	GCA_945643635.2	[41]
*Caenorhabditis bovis*	Free-living	GCA_902829315.1	[56]
*Caenorhabditis briggsae*	Free-living	GCA_022453885.1	[57]
*Caenorhabditis elegans*	Free-living	GCF_000002985.6	[16,51,52]
*Caenorhabditis inopinata*	Free-living	GCA_003052745.1	[58]
*Caenorhabditis nigoni*	Free-living	GCA_002742825.1	[59]
*Caenorhabditis remanei*	Free-living	GCF_010183535.1	[60]
*Caenorhabditis tropicalis*	Free-living	GCA_000186765.1	[16,51,52]
*Diploscapter coronatus*	Free-living	GCA_002207785.1	[61]
*Mesorhabditis belari*	Free-living	GCA_958450365.1	[62]
*Oscheius tipulae*	Free-living	GCA_013425905.1	[63]
*Pristionchus exspectatus*	Free-living	GCA_911812115.1	[64]
*Pristionchus pacificus*	Free-living	GCA_000180635.4	[65]

Orthology inference was performed using OrthoFinder in ultrasensitive mode, and orthogroups were queried for homologs of intrinsic apoptosis pathway components defined in *Caenorhabditis elegans* and *Haemonchus contortus* [1,8–10,12,13], including CEP-1, EGL-1, CED-13, CED-9, CED-4, CED-3, DRE-1, and PUF-8 (Table S2); a summary of the orthogroups is given in Table S3. Because sequence-based orthology inference depends on accurate gene annotation, genome-guided searches were performed to identify homologs that may not have been annotated. Homologs were identified using FastProteinExonerate (https://github.com/stroehleina/FastProteinExonerate). In short, protein sequences from *Caenorhabditis elegans* and *Haemonchus contortus* were mapped to genomic assemblies using pblat [19], and corresponding genomic regions, including 500-bp flanking sequences, were extracted using BEDTools v2.31.1 [20]. These regions were analyzed using Exonerate v2.4.0 [21] to predict coding sequences. To maximize sensitivity, an iterative approach was employed in which homologs identified in initial OrthoFinder and FastProteinExonerate analyses were used as queries in subsequent rounds of genome mapping and FastProteinExonerate prediction. An overview of putative homologs identified using OrthoFinder or FastProteinExonerate is provided in Table S4. Failure to recover a homolog was treated as nondetection rather than definitive evidence of biological absence.

Highly divergent homologs of the BH3-only protein CED-13 were not recovered using OrthoFinder or FastProteinExonerate. We therefore performed an additional structure-based search using Foldseek [22] where sufficiently extensive pre-computed structural datasets were available. Pre-computed protein structure models were obtained from the AlphaFold Protein Structure Database [23] for nematodes with more than 10,000 available protein models (Table S1) and used to construct Foldseek databases. This criterion permitted structure-based searches for 14 of the 21 included species. CED-13 of *Caenorhabditis elegans* (NP_509402.1) was modeled separately using AlphaFold 3 [24] and used as the Foldseek query.

### Characterization of apoptosis pathway homologs

Following identification, putative apoptosis pathway homologs were characterized using conserved-domain analyses, manual curation, phylogenetics, transcriptomics, and structural comparisons. To exclude false-positive identifications, putative homologs recovered using OrthoFinder and FastProteinExonerate were screened for conserved domains using HMMER v3.4 [25], based on the domain models listed in Table S5. The Pfam model [26] for detecting a conserved EGL-1 domain (PF11430.13) contains 22 sequences, all of which belong to the genus *Caenorhabditis* and did not reliably detect conserved domains in putative EGL-1-like homologs of clade V nematodes. We generated an expanded PF11430 model by running HMMsearch on all metazoan proteins (*n* = 41,497,433) available in the UniProt database (accessed on 2026 March 30) using an *e*-value threshold of 10. In addition to the protein sequences of *Caenorhabditis* spp., this model included sequences from other clade V nematodes (*Cylicocyclus nassatus*, *Mesorhabditis belari*, and *Pristionchus* spp.), the clade IV nematode *Bursaphelenchus xylophilus*, and arthropods, chordates, and mollusks (Table S6). We also added EGL-1-like homologs of clade V nematodes identified and confirmed using the original PF11430.13 model (*n* = 23). We then iteratively searched the remaining unconfirmed putative EGL-1-like homologs in 2 rounds, confirming additional hits (*n* = 11 and *n* = 1, respectively; Table S6). Since including more sequences reduced the length of the domain, the profile hidden Markov model (HMM) was updated only after it identified the last hit and did not include that hit. Model specificity was evaluated using a randomized-sequence control: for each identified EGL-1-like sequence, 100 shuffled sequences were generated using EMBOSS shuffleseq, retaining sequence length and amino acid composition while disrupting residue order, and searched with the expanded model. As an additional validation, the complete proteomes of the included clade V species (463,639 proteins) were searched with the expanded model. These controls were used to assess specificity and susceptibility to composition-driven matches; detection by the expanded profile HMM alone was not considered evidence of homology. The expanded PF11430.hmm is given in File S1, and construction and validation results are reported in Table S6. The reported *e*-values are domain independent. For PUM-1/PUM-2-like homologs, the mean *e*-values of the 8 identified domains are given.

Manual curation was performed for species in which more than one putative homolog was identified or when putative homologs did not contain a match for all conserved domains. Sequences obtained from OrthoFinder and FastProteinExonerate were aligned using MUSCLE v3.8.1551 [27] and subsequently selected based on sequence similarity. Some homologs were annotated as fragmented proteins containing only a subset of the expected conserved domains. Hits from FastProteinExonerate contained all conserved domains and were used for subsequent analysis (File S2). Homologs were then classified as orthologs (single-copy genes) or paralogs (multicopy genes), except where highly similar duplicate gene models could not be distinguished confidently from assembly or haplotypic redundancy.

Following confirmation and curation, phylogenetic relationships among homologs were inferred using conserved-domain sequences. Sequences were aligned to the respective profile HMM (see Table S5) using HMMER v3.4 to obtain sequences of the conserved domains. Subsequently, aligned sequences were trimmed with ClipKIT [28] in default smart-gap mode, concatenated, and used to generate protein trees employing IQ-TREE v3.1.1 [29] using ModelFinderPlus [30]. Node support was calculated using 1,000 ultrafast bootstrap replicates [31] and 1,000 Shimodaira–Hasegawa-like approximate likelihood-ratio tests [32]. Trees were rooted using *Caenorhabditis elegans* proteins and visualized in R v4.5.3 using ggtree [33], with tree data processed using treeio v1.34.0 [34].

To assess transcriptional support for identified homologs, publicly available RNA sequencing (RNA-seq) datasets were retrieved using SRA Toolkit v3.2.1 (https://github.com/ncbi/sra-tools) (Table 2 and Table S7). Raw reads were assessed for quality using FastQC v0.12.1 (https://www.bioinformatics.babraham.ac.uk/projects/fastqc/) and trimmed using Trimmomatic v0.40 [35]. Reads were aligned to reference genomes using HISAT2 v2.2.2 [36], and the resulting SAM files were converted to sorted and indexed BAM files using SAMtools v1.18 [37]. For species represented by multiple developmental stages, BAM files were pooled only to maximize sensitivity for determining whether the transcription of a candidate homolog was detectable in any available stage; the pooled values were not used to infer stage-specific expression. Gene-level counts were obtained using featureCounts v2.0.6 [38] and normalized to transcripts per million (TPM). TPM percentiles were calculated within each species-specific dataset for visualization only and were not interpreted as quantitatively comparable measures of expression among species, laboratories or developmental stages. For *Caenorhabditis elegans*, *Caenorhabditis nigoni*, *Haemonchus contortus*, and *Necator americanus*, gene-level TPM values were manually linked to the protein accessions corresponding to the longest annotated isoforms represented in the respective proteomes. Transcriptional support could not be assessed reliably for *Ancylostoma duodenale* (20,013 transcripts, 77% with TPM values of zero), *Angiostrongylus vasorum* (152 detected transcripts), *Caenorhabditis bovis* (no transcriptomic dataset available), or *Caenorhabditis briggsae* (805 transcripts, 74% with TPM values of zero). RNA-seq data were available for *Ostertagia ostertagi* but could not be incorporated into the gene-level analysis because the corresponding annotation features required for read assignment were unavailable from the NCBI at the time of the study.

**Table 2.** RNA-seq datasets included in this study. Listed are species, lifestyle, life cycle stage, and reference. A detailed description including SRA accession numbers is given in Table S7.

Species	Lifestyle	Life cycle stages	Reference
*Ancylostoma duodenale*	Parasitic	Adult	[47]
*Angiostrongylus vasorum*	Parasitic	Not defined	[48]
*Cylicocyclus nassatus*	Parasitic	Adult	[49]
*Haemonchus contortus*	Parasitic	Egg, L1, L2, L3, L4, adult	[66]
*Necator americanus*	Parasitic	L3, adult	[67]
*Nippostrongylus brasiliensis*	Parasitic	L3, L4, adult	[53,68]
*Ostertagia ostertagi*	Parasitic	N/I	N/I
*Teladorsagia circumcincta*	Parasitic	L3, adult	[55]
*Allodiplogaster sudhausi*	Free-living	Adult	[41]
*Caenorhabditis bovis*	Free-living	N/A	N/A
*Caenorhabditis briggsae*	Free-living	Embryo, L1, L2, L3, L4, adult	[69]
*Caenorhabditis elegans*	Free-living	Embryo, L1, L4, adult, dauer	[69,70]
*Caenorhabditis inopinata*	Free-living	L3, L4, adult	[71]
*Caenorhabditis nigoni*	Free-living	Adult	[72]
*Caenorhabditis remanei*	Free-living	L4 to adult	[73]
*Caenorhabditis tropicalis*	Free-living	Embryo, L4, adult	[74]
*Diploscapter coronatus*	Free-living	Egg	[75]
*Mesorhabditis belari*	Free-living	L1, adult	[76]
*Oscheius tipulae*	Free-living	Embryo, L1, L2, L3, L4, adult, dauer	[77]
*Pristionchus exspectatus*	Free-living	0, 12, 24, 48, 60, and 72 h of development	[78]
*Pristionchus pacificus*	Free-living	0, 24, 48, and 72 h of development	[78]

RNA-seq, RNA sequencing; SRA, Sequence Read Archive; N/A, not available; N/I, not included

### Structural analyses of apoptosis pathway homologs

Protein sequences of confirmed homologs were analyzed using SignalP 6.0 [39] to identify N-terminal signal peptides that could affect subsequent structural comparisons; where a signal peptide was predicted, the sequence was cleaved after the predicted cleavage site before modeling. CASP-3-like (CED-3) homologs were cleaved at the start of the identified caspase domain (PF00656.28). Structures of the pathway homologs analyzed in this study were then modeled using AlphaFold 3 [24].

Structural similarity was assessed by comparing predicted protein structures with those of well-characterized proteins from *Caenorhabditis elegans* [1,8,10,12] and recently identified homologs from *Haemonchus contortus* [13] using TM-align v20240303 [40]. In addition, homologs representing all parasitic clade V nematodes were compared with one another and were also compared with corresponding homologs from host animals: *Bos taurus* (*Ostertagia ostertagi*), *Canis lupus familiaris* (*Angiostrongylus vasorum*), *Equus caballus* (*Cylicocyclus nassatus*), *Homo sapiens* (*Ancylostoma duodenale* and *Necator americanus*), *Ovis aries* (*Haemonchus contortus* and *Teladorsagia circumcincta*), and *Rattus norvegicus* (*Nippostrongylus brasiliensis*). For species containing multiple homologs of a component, structural-similarity values were first summarized within species so that paralogs were not treated as independent species-level observations. For each pathway component, we then summarized the median structural similarity among parasitic nematodes, the median similarity between parasites and their corresponding hosts, the difference between these values, and the consistency of the direction of the comparison across evaluable parasite species. Formal hypothesis testing was not applied because the pairwise structural comparisons, multiple homologs within some species, and phylogenetic relatedness among species violate independence assumptions.

## Results

### Execution module homologs are relatively conserved across clade V nematodes

Orthology inference across 20 included clade V nematodes identified 32,632 orthogroups. Of these, 3,449 were represented in all species, providing the framework for the focused comparative analysis of intrinsic-apoptosis-associated homologs. We did not attribute the broader set of nonuniversal orthogroups to particular functions or lineages because such interpretation would require a dedicated analysis that accounts for phylogenetic sampling and variation in genome and annotation completeness.

Interrogation of orthogroups corresponding to BCL-2-like, APAF-1-like, and CASP-3-like proteins revealed consistent clustering of these candidate execution module components in shared orthogroups across the included species (Table S3). Homologs were recovered from most species: a BCL-2-like homolog was not detected in *Mesorhabditis belari*, and detection of the BCL-2-like homolog in *Pristionchus pacificus* required additional genome-guided searches (Table S4). APAF-1-like and CASP-3-like homologs were recovered from all included species. No caspase activation and recruitment (CARD) domains were identified in the putative APAF-1-like homologs of the free-living nematodes *Allodiplogaster sudhausi* and *Oscheius tipulae*. Apart from these exceptions, identified homologs were supported by the expected conserved-domain architecture and, where suitable RNA-seq data were available, by detectable transcription (Fig. 1 and Table S9). No WD40 domains were detected in any APAF-1-like homolog identified in this study. These observations establish conservation of homologous proteins and structural features but do not demonstrate equivalent pathway function in every species.

**Fig. 1.**
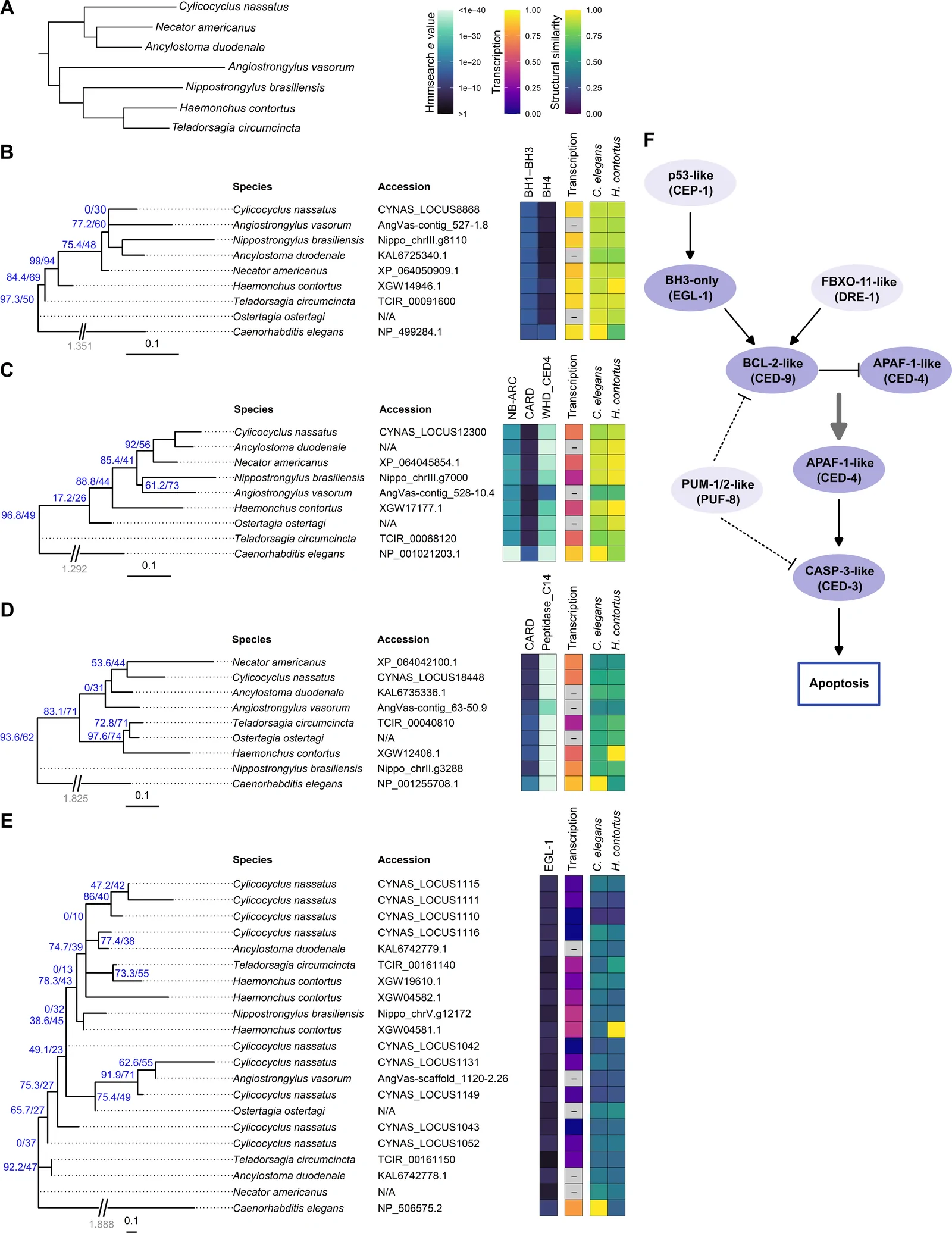
Candidate execution module and BH3-only homologs in parasitic clade V nematodes. Panel (A) shows the relevant section of the species tree generated using OrthoFinder [17,18]. Panels (B) to (D) show homologs corresponding to the proposed execution module: (B) BCL-2-like, (C) APAF-1-like, and (D) CASP-3-like. Panel (E) shows the BH3-only regulator EGL-1-like proteins. Phylogenetic trees were inferred from concatenated conserved domains for each protein. Node support values are shown in blue as Shimodaira–Hasegawa-like approximate likelihood-ratio test (SH-aLRT)/ultrafast bootstrap (UFBoot); values associated with truncated *Caenorhabditis elegans* branches indicate the complete branch length, while the scale bar represents the remaining branch lengths. The tree was rooted using *Caenorhabditis elegans* proteins. Conserved domains identified using HMMsearch are presented as domain-independent *e*-values. Transcription is shown as within-species percentile values for visualization only (dashes indicate that no suitable transcriptomic data were available) and is not interpreted quantitatively across species. Structural similarity of full-length homologs to proteins from *Caenorhabditis elegans* and *Haemonchus contortus* is shown as TM-align values. Panel (F) shows the intrinsic apoptosis pathway previously proposed for *Haemonchus contortus* [13] as a biological reference framework; it was not inferred *de novo* for all species in the present study. Proteins analyzed here are highlighted using a darker color.

Duplicate gene models corresponding to all 3 execution module components were identified in *Allodiplogaster sudhausi*, consistent with previous evidence for a recent whole-genome duplication in this lineage [41]. *Diploscapter coronatus* also contained 2 highly similar gene models for each of BCL-2-like, APAF-1-like, and CASP-3-like proteins (with 97.5% to 99.6% sequence identity). However, its high proportion of duplicated BUSCOs (genome, 67.9%; proteome, 88.9%) indicates substantial redundancy in the available assembly and annotation, so these copies were treated conservatively as highly similar duplicate gene models rather than as confirmed paralogs or evidence of a lineage-specific expansion. *Pristionchus* spp. showed lineage-specific expansions of BCL-2-like and APAF-1-like homologs. We also aligned clade V BCL-2-like homologs to BCL-2 and pro-apoptotic BAX, BAK, and BOK proteins from *Homo sapiens* and *Mus musculus*; the nematode BCL-2-like homologs clustered together, separately from the mammalian BAX, BAK, and BOK proteins and grouped with mammalian BCL-2 (Fig. S1).

For the BH3-only regulator EGL-1, putative homologs were identified in most included clade V nematodes, except *Allodiplogaster sudhausi* and *Diploscapter coronatus*. Detection in *Necator americanus* required additional genome-guided searches (Table S4). EGL-1-like homologs exhibited substantially greater variability than the candidate execution module proteins, with sequences from *Caenorhabditis elegans* and *Haemonchus contortus* clustering into separate orthogroups (Table S3). Detection of a conserved EGL-1 domain required the expanded PF11430 model (Table S6). In the randomized-sequence validation, HMMsearch of 100 shuffled sequences per identified EGL-1-like sequence yielded no hits at the threshold used (observed hit rate, 0%). A proteome-wide search of 463,639 proteins recovered almost all EGL-1-like homologs identified independently by the orthology/genome-guided workflow and identified 25 additional candidate proteins (Table S6). These results support the specificity of the expanded model under the controls used but do not independently establish biological homology or function for every marginal candidate. Where suitable RNA-seq data were available, candidate EGL-1-like homologs showed detectable transcription but limited structural similarity to *Caenorhabditis elegans* or *Haemonchus contortus* proteins (Fig. 1E and Table S9). Multiple EGL-1-like copies occurred in *Haemonchus contortus*, *Ancylostoma duodenale*, *Teladorsagia circumcincta*, and particularly *Cylicocyclus nassatus*. SignalP analysis identified a predicted signal peptide only for the EGL-1-like protein of *Caenorhabditis tropicalis*; no signal peptide was detected in the CASP-3-like homologs analyzed.

*Caenorhabditis elegans* CED-13 [9] formed a singleton OrthoFinder orthogroup, and no additional CED-13-like homolog was identified in the 20 other included species using genome-guided FastProteinExonerate searches. Foldseek provided an additional structure-based search for the 14 species with sufficiently extensive pre-computed structural coverage. Structural searches could not be performed for *Angiostrongylus vasorum*, *Ostertagia ostertagi*, *Allodiplogaster sudhausi*, *Caenorhabditis inopinata*, *Diploscapter coronatus*, *Oscheius tipulae*, or *Pristionchus exspectatus*. In the available structural databases, the *Caenorhabditis elegans* CED-13 query was recovered with an *e*-value of 1 × 10^−12^, whereas the best-hit *e*-values for other species ranged from 0.5 to 2.6 (Table S11), consistent with random matches. Thus, no additional CED-13-like homolog was detected by the sequence-, genome-, or, where available, structure-based approaches used here; highly divergent homologs may nevertheless remain beyond their detection limits.

### Upstream regulators show variation across lineages

Regulatory proteins associated with intrinsic apoptosis showed heterogeneous evolutionary patterns. FBXO-11-like and PUM-1/PUM-2-like homologs clustered consistently into orthogroups, whereas p53-like homologs from *Caenorhabditis elegans* and *Haemonchus contortus* occurred in distinct orthogroups (Table S3). These patterns indicate comparatively stable homology assignment for FBXO-11-like and PUM-1/PUM-2-like proteins but do not establish that their biological roles are restricted to, or conserved within, apoptosis.

FBXO-11-like homologs were identified in all included species except *Pristionchus exspectatus*. Candidate identities were supported by conserved-domain architecture, structural similarity, and, where suitable datasets were available, detectable transcription (Fig. 2B and Table S10). Most nematodes had a single FBXO-11-like homolog, whereas 2 paralogs were identified in *Teladorsagia circumcincta*. *Diploscapter coronatus* contained 2 gene models encoding FBXO-11-like proteins whose predicted amino acid sequences were 99.8% identical.

**Fig. 2.**
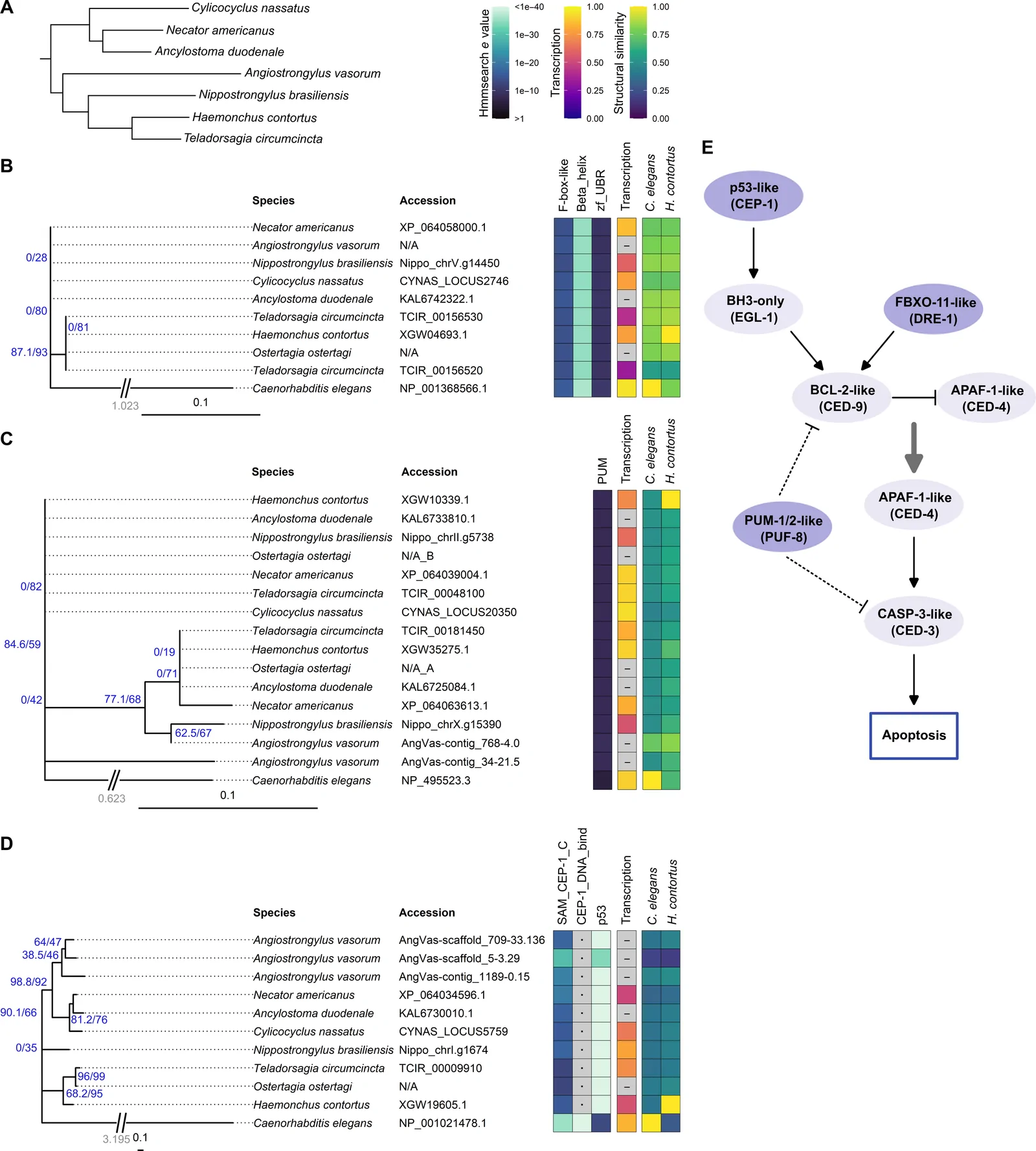
Candidate upstream regulators associated with intrinsic apoptosis in parasitic clade V nematodes. Panel (A) shows the relevant section of the species tree generated using OrthoFinder [17,18]. Panels (B) to (D) show homologs of upstream regulators: (B) FBXO-11-like (DRE-1), (C) PUM-1/PUM-2-like (PUF-8), and (D) p53-like (CEP-1). Phylogenetic trees were inferred from concatenated conserved domains for each protein. Node support values are shown in blue as Shimodaira–Hasegawa-like approximate likelihood-ratio test (SH-aLRT)/ultrafast bootstrap (UFBoot); values associated with truncated *Caenorhabditis elegans* branches indicate the complete branch length, while the scale bar represents the remaining branch lengths. The tree was rooted using *Caenorhabditis elegans* proteins. Conserved domains identified using HMMsearch are presented as domain-independent *e*-values (dots indicate that no conserved domain was detected). Transcription is shown as within-species percentile values for visualization only (dashes indicate that no suitable transcriptomic data were available) and is not interpreted quantitatively across species. Structural similarity of full-length homologs to proteins from *Caenorhabditis elegans* and *Haemonchus contortus* is shown as TM-align values. Panel (E) shows the intrinsic apoptosis pathway previously proposed for *Haemonchus contortus* [13] as a biological reference framework; it was not inferred *de novo* for all species in the present study. Proteins analyzed here are highlighted using a darker color.

PUM-1/PUM-2-like homologs were identified in all included clade V nematodes and were supported by the detection of 8 conserved PUM domains, structural similarity, and, where evaluable, detectable transcription (Fig. 2C and Table S10). Gene duplication was common, with 2 paralogs present in most parasitic species, except for *Cylicocyclus nassatus*, which had a single ortholog (Table S10).

p53-like homologs were detected in most included species but not in *Diploscapter coronatus*; detection in the free-living nematode *Pristionchus pacificus* required a combination of proteome-guided and genome-guided searches (Table S4). In parasitic nematodes, identified homologs contained both the SAM_CEP-1_C and p53 DNA-binding domains, whereas the CEP-1-DNA_bind domain was detected only in free-living nematodes of the genus *Caenorhabditis*. Single orthologs were identified for most parasitic nematodes, with lineage-specific duplications in *Angiostrongylus vasorum*. Structural analyses and, where suitable RNA-seq datasets were available, detectable transcription supported the identification of these homologs (Fig. 2D and Table S10). Across all pathway components, transcriptional evidence was used only as support that a candidate gene model was transcribed; the heterogeneous public datasets were not used to compare expression quantitatively among species or developmental stages.

### Structural comparisons inform candidate target prioritization

Structural comparisons revealed high similarity among parasitic clade V nematode homologs of the candidate execution module proteins BCL-2-like, APAF-1-like, and CASP-3-like (Fig. 3 and Table 3 ). The median TM-align scores among parasitic nematodes were 0.90, 0.94, and 0.92, respectively, compared with median parasite–host scores of 0.62, 0.47, and 0.80, corresponding to differences of 0.28, 0.47, and 0.12. Greater within-parasite structural similarity was observed in all 8 evaluable parasite species for each of these 3 execution module homologs, although the magnitude of the median difference was smaller for CASP-3-like proteins. Among regulators, FBXO-11-like homologs were comparatively well conserved among parasites (median 0.80), PUM-1/PUM-2-like homologs showed intermediate conservation (0.59), and p53-like and EGL-1-like homologs were more divergent (0.41 and 0.38, respectively). The corresponding parasite–host medians were 0.77, 0.52, 0.40, and 0.36, and, although less prominent, the direction of parasite-versus-host differences was greater for all FBXO-11-like and PUM-1/PUM-2-like proteins and for most p53-like and EGL-1-like homologs. These global structural comparisons identify differences in evolutionary conservation that can inform target prioritization, but they do not establish druggability, binding-site selectivity, or therapeutic safety.

**Fig. 3.**
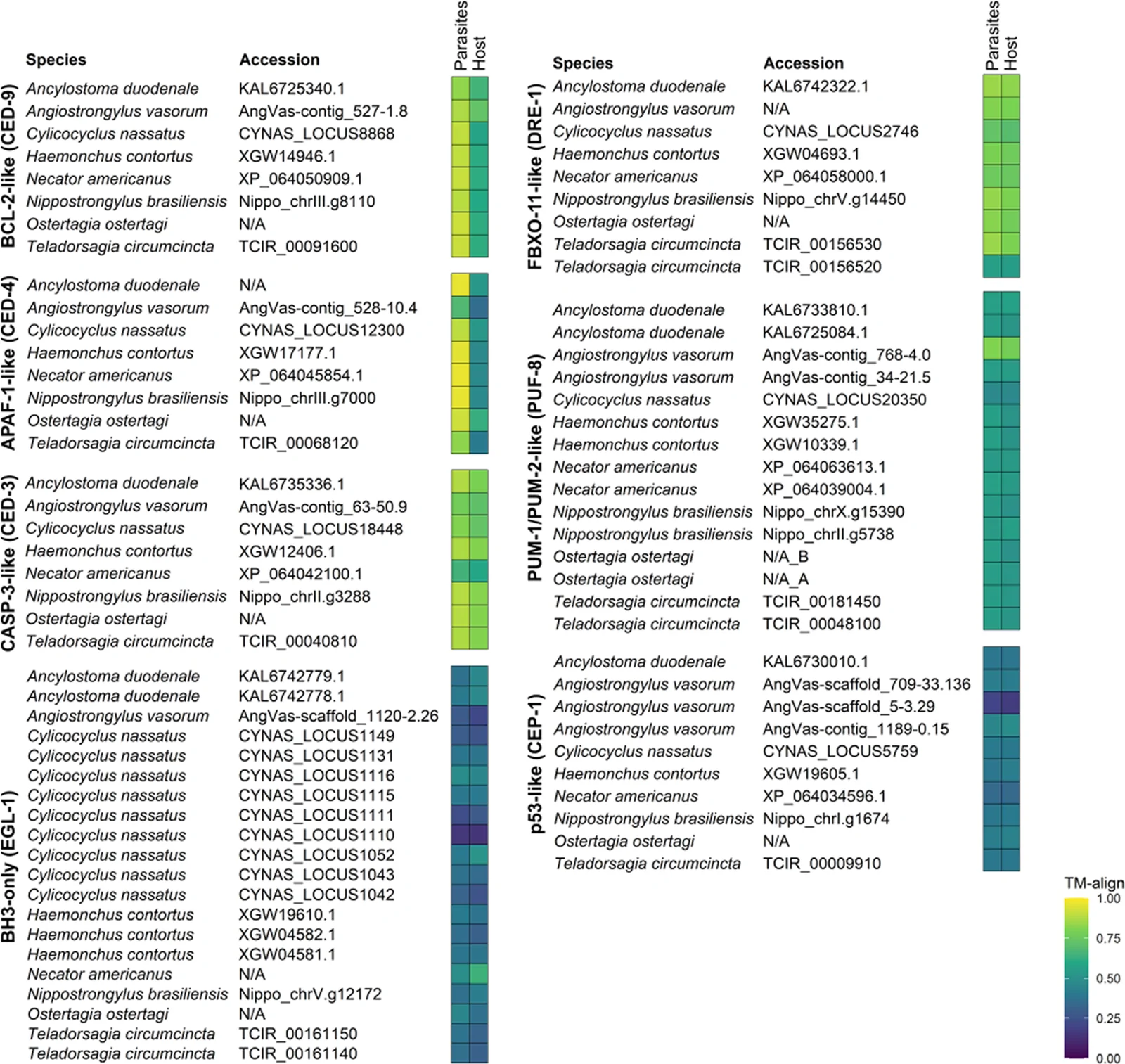
Structural similarity of intrinsic-apoptosis-associated homologs in parasitic clade V nematodes and their corresponding hosts. Structural similarity was assessed using TM-align for full-length homologs of most proteins; CASP-3-like homologs were cleaved at the start of the identified caspase domain (PF00656.28). For each parasite homolog, the figure shows structural similarity to homologs from other parasitic clade V nematodes and to the corresponding homolog from its respective host. These comparisons are distinct from the *Caenorhabditis elegans* and *Haemonchus contortus* reference alignments shown in Figs. 1 and 2. Species-level descriptive summaries used for interpretation, including medians and within-parasite versus parasite–host differences, are reported in Table 3.

**Table 3.** Overview of intrinsic-apoptosis-associated homologs in clade V nematodes. Recovery is presented as the number of included species in which homologs were identified. For each component, structural similarity is summarized descriptively at the parasite-species level as the median TM-align score among parasitic nematodes and the median score between each parasite and its corresponding host, together with the difference between these values and the direction of the comparison across evaluable parasite species. Where multiple homologs occurred within a species, values were first summarized within that species. Formal inferential tests were not applied because pairwise structural comparisons, within-species paralogs, and phylogenetic relatedness violate independence assumptions.

Component	Recovery	Median TM-align (within parasites)	Median TM-align (parasite–host)	Difference	Direction across parasite species
BCL-2-like (CED-9)	20/21	0.90	0.62	0.28	Greater in 8/8
APAF-1-like (CED-4)	21/21	0.94	0.47	0.47	Greater in 8/8
CASP-3-like (CED-3)	21/21	0.92 ^a^	0.80 ^a^	0.12	Greater in 8/8
BH3-only (EGL-1)	19/21	0.38	0.36	0.02	Greater in 5/8
p53-like (CEP-1)	20/21	0.41	0.40	0.01	Greater in 7/8
FBXO-11-like (DRE-1)	20/21	0.80	0.77	0.03	Greater in 8/8
PUM-1/PUM-2-like (PUF-8)	21/21	0.59	0.52	0.06	Greater in 8/8

^a^ CASP-3-like proteins were truncated proteins cleaved at the start of the identified caspase domain (PF00656.28).

## Discussion

Intrinsic apoptosis is widely regarded as a conserved cellular process, yet the present comparative analyses show that candidate pathway components in clade V nematodes differ markedly in evolutionary conservation, gene-copy pattern, and detectability. By integrating orthology inference, genome-guided searches, and structure-informed analyses, we identified a comparatively conserved set of BCL-2-like, APAF-1-like, and CASP-3-like homologs corresponding to the proposed execution module, whereas the associated regulatory architecture was heterogeneous. Importantly, these conclusions concern homology, structural conservation, and candidate pathway placement; they do not demonstrate that the proteins retain identical biological functions, interaction partners, or mechanisms in every species. Experimental perturbation, interaction, and biochemical studies will be required to establish those functions directly.

These findings extend previous work on apoptosis in nematodes in a way that is conceptually broader than simply increasing taxon sampling. A phylum-wide analysis by Young *et al.* [2] demonstrated extensive variation in BCL-2 family proteins and adaptor components across nematode clades, indicating that the *Caenorhabditis elegans* pathway does not represent a universal model. More recently, pathway reconstruction in *Haemonchus contortus* identified candidate components of intrinsic apoptosis in a single parasitic clade V species and suggested regulatory divergence [13]. The present study tests which features of that reconstruction extend across a resource-defined clade V dataset. BCL-2-like, APAF-1-like, and CASP-3-like proteins showed consistent orthology and relatively high structural conservation, whereas regulatory components differed substantially in orthology assignment, structural conservation, copy number, and detectability. The comparative design also enabled direct descriptive assessment of structural conservation among parasitic nematodes relative to their corresponding host homologs. Because taxon selection was resource driven rather than phylogenetically balanced, these clade-wide patterns should be interpreted with appropriate caution.

The regulatory architecture cannot be reduced to a uniformly divergent second layer. EGL-1-like homologs were especially variable, with inconsistent orthology assignment, relatively low structural conservation, and frequent copy-number expansion. Such variation is compatible with greater evolutionary flexibility in this BH3-only regulator and could permit lineage-specific subfunctionalization or neofunctionalization, although the functions and interaction specificities of the individual copies remain to be established experimentally. By contrast, FBXO-11-like homologs were comparatively well conserved, and PUM-1/PUM-2-like homologs showed intermediate conservation. Both protein families have biological roles beyond apoptosis, so their conservation cannot be attributed solely to their proposed contribution to apoptotic regulation. p53-like homologs showed inconsistent orthology assignment and lower structural conservation. *Caenorhabditis elegans* CED-13 was not accompanied by a detectable homolog in the other species using the approaches applied here, but the singleton orthogroup and negative genome/structure searches do not demonstrate biological absence.

Gene-copy patterns provided an additional, distinct dimension of pathway evolution. The principal execution module homologs were less extensively expanded than EGL-1-like proteins, but they were not universally single-copy. Previous genomic evidence supports a recent whole-genome duplication in *Allodiplogaster sudhausi* [41], providing a plausible explanation for duplicate execution module gene models in that species, whereas the highly similar duplicate models in *Diploscapter coronatus* were interpreted cautiously because its BUSCO profile indicates substantial assembly/proteome redundancy. Lineage-specific expansions of BCL-2-like and APAF-1-like homologs were also observed in *Pristionchus* spp. In contrast, EGL-1-like homologs showed more frequent and sometimes larger expansions, including in *Haemonchus contortus*, *Ancylostoma duodenale*, *Teladorsagia circumcincta*, and *Cylicocyclus nassatus*. One possible explanation is that stronger constraints on molecular interactions limit diversification of execution module components, whereas regulatory components tolerate greater copy-number flexibility; this remains a hypothesis rather than a demonstrated functional mechanism.

Standard sequence-based homology approaches recovered most candidate pathway components, but additional genome-guided searches were required for individual taxa, and the expanded EGL-1 HMM improved the recovery of highly divergent candidate homologs. The shuffled-sequence and proteome-wide controls supported the specificity of the expanded model under the conditions tested while also emphasizing that detection by a sequence model is not equivalent to functional validation. More generally, nondetection is itself influenced by evolutionary divergence and technical sensitivity [42,43], as well as by genome and annotation quality, and should not be equated automatically with biological absence. This consideration is especially important when reconstructing rapidly evolving biological systems and when transferring functional annotations across homologs [44]. At the same time, the lack of a clear relationship between BUSCO completeness and recovery of the 7 principal pathway-associated components argues against genome/proteome incompleteness as a simple explanation for the observed pattern: for example, *Angiostrongylus vasorum* had the lowest proteome BUSCO completeness among species with this metric available (68.8%), yet all 7 components were recovered, whereas several species with substantially higher completeness lacked one or more detectable homologs. Integrative sequence-, genome-, and structure-based evidence therefore provides a stronger basis for comparative prioritization than any single detection method.

The structural comparisons refine, rather than establish, the translational argument for apoptosis-associated proteins. Conservation of a candidate protein among parasitic nematodes may favor broad-spectrum applicability, whereas divergence from the corresponding host homolog may provide opportunities for parasite-selective targeting; these are separate considerations. BCL-2-like and APAF-1-like homologs combined high structural conservation among parasites with larger differences from their host homologs than were observed for CASP-3-like proteins, making them particularly relevant candidates for further investigation. However, global TM-align similarity does not establish druggability, binding-site selectivity, or therapeutic safety, and the 3 execution module components should not be treated as equivalent on those grounds. Conversely, the extensive divergence of EGL-1-like and p53-like proteins among parasite lineages does not make them intrinsically undruggable but may reduce their suitability as broad-spectrum clade V targets. Recent work illustrates how molecular-resolution datasets can support candidate prioritization in *Haemonchus contortus* [45] and how computational and structural predictions can be advanced to experimental tests of biological function in nematode proteins [46]. For the apoptosis-associated candidates identified here, comparable biochemical, cellular, and *in vivo* validation will be required before translational potential can be established.

These findings should be interpreted in light of several methodological and evidentiary considerations. First, all analyses were computational and therefore did not provide direct functional validation of pathway activity, protein interactions, or regulatory mechanisms. Second, failure to detect a homolog cannot be interpreted as unequivocal evidence of biological absence: incomplete or fragmented genomic sequence, imperfect gene annotation, and sequence divergence beyond the detection thresholds of the methods used could each prevent recovery of a genuine homolog, and a relevant function could also be performed by a protein not identifiable through homology to the reference proteins examined here. Genome and proteome BUSCO metrics reduced, but did not eliminate, these uncertainties. Third, the resource-driven taxon set was not phylogenetically balanced, and the overrepresentation of *Caenorhabditis* affects interpretation of broad clade-wide descriptive patterns. Fourth, heterogeneous public RNA-seq datasets supported only the presence of detectable transcription and were not suitable for quantitative cross-species or stage-by-stage expression comparisons. Finally, structural modeling and global structural similarity provide evidence about protein architecture, not functional equivalence or pharmacological selectivity. Experimental validation will therefore be required to test the proposed pathway roles and target potential of individual proteins.

## Conclusion

Candidate intrinsic-apoptosis-associated proteins in clade V nematodes show a comparatively conserved BCL-2-like:APAF-1-like:CASP-3-like execution module embedded within a heterogeneous regulatory architecture. Regulatory components differ substantially in orthology, structural conservation, detectability, and gene-copy pattern, rather than forming a uniformly divergent layer. The conservation of execution module homologs among parasitic nematodes, together with component-specific differences from host homologs, supports their prioritization for experimental investigation but does not itself establish conserved function, druggability, or selectivity. These findings provide a framework for comparative studies of apoptosis in other nematode clades and for experimentally testing its potential as an axis for anthelmintic discovery.

## Data Availability

The data that support the findings of this study are included in the article or the Supplementary Materials. Alignment files used to generate protein trees, as well as the corresponding tree files and TM values, have been deposited in Zenodo (10.5281/zenodo.23356255). The script used for the analysis of RNA-seq datasets is available via GitHub (https://github.com/vetscience/rnaseq_tpm_pipeline).

## Abbreviations

**Protein abbreviations**

APAF-1  Apoptotic protease-activating factor 1
BAK   BCL-2 antagonist/killer
BAX   BCL-2-associated X protein
BCL-2  B-cell lymphoma 2
BOK   BCL-2-related ovarian killer
CASP-3  Caspase 3
CED-3  Cell death abnormality 3
CED-4  Cell death abnormality 4
CED-9  Cell death abnormality 9
CED-13  Cell death abnormality 13
CEP-1  *Caenorhabditis elegans* p53-like protein 1
DRE-1  Daf-12 redundant with 1
EGL-1  Egg-laying defective 1
FBXO-11  F-box only protein 11
PUF-8  Pumilio/FBF domain-containing 8
PUM-1  Pumilio homolog 1
PUM-2  Pumilio homolog 2

**Other abbreviations**

CARD  Caspase activation and recruitment
HMM   Hidden Markov model
MOMP  Mitochondrial outer membrane permeabilization
RNA-seq  RNA sequencing
TPM   Transcripts per million
