# Supplementary material for: Comparative Reconstruction of Intrinsic Apoptosis in Clade V Nematodes Reveals a Conserved Execution Module and Heterogeneous Regulatory Architecture
Source: Comput Struct Biotechnol J. 2026 Nov 13;35(1):0239. doi: 10.34133/csbj.0239 (PMC13760059; doi:10.34133/csbj.0239)
Supplement: Supplementary file 1 [file csbj.0239.f1.zip › Fig S1.docx]

**SUPPLEMENTARY FIGURE**

**Fig. S1. Phylogenetic placement of clade V nematode BCL-2–like homologues relative to mammalian BCL-2, BAX, BAK and BOK proteins.** A maximum–likelihood tree was inferred from aligned conserved BH1-BH3 and BH4 domain sequences of the nematode homologues and reference proteins from Homo sapiens and Mus musculus. Species names and accession identifiers are shown at the tips, and the BCL-2**–like group, as well as the** mammalian **BCL-2**, BAX, BAK and BOK groups are indicated at the right. Blue node labels report SH-aLRT/UFBoot support values, in that order, based on 1,000 replicates each. The tree was rooted using Homo sapiens BAX (Q07812), and the scale bar represents amino-acid substitutions per site.
